# Avoiding a reproducibility crisis in regulatory toxicology—on the fundamental role of ring trials

**DOI:** 10.1007/s00204-024-03736-z

**Published:** 2024-04-30

**Authors:** Miriam N. Jacobs, Sebastian Hoffmann, Heli M. Hollnagel, Petra Kern, Susanne N. Kolle, Andreas Natsch, Robert Landsiedel

**Affiliations:** 1 Radiation, Chemical and Environmental Hazards (RCE), Department of Toxicology, UK Health Security Agency (UKHSA), Harwell Science and Innovation Campus, Chilton, OX11 0RQ UK; 2 Seh Consulting + Services, Paderborn, Germany; 3 Dow Europe GmbH, Horgen, Switzerland; 4 Procter & Gamble Services Company NV, Strombeek-Bever, Belgium; 5 BASF SE, Experimental Toxicology and Ecology, Ludwigshafen am Rhein, Germany; 6 Givaudan Suisse SA, 8310 Kemptthal, Switzerland; 7 Free University of Berlin, Biology, Chemistry and Pharmacy, Pharmacology and Toxicology, Berlin, Germany

**Keywords:** Validation, Ring trials, OECD Test Guidelines, Robustness, Reliability

## Abstract

The ongoing transition from chemical hazard and risk assessment based on animal studies to assessment relying mostly on non-animal data, requires a multitude of novel experimental methods, and this means that guidance on the validation and standardisation of test methods intended for international applicability and acceptance, needs to be updated. These so-called new approach methodologies (NAMs) must be applicable to the chemical regulatory domain and provide reliable data which are relevant to hazard and risk assessment. Confidence in and use of NAMs will depend on their reliability and relevance, and both are thoroughly assessed by validation. Validation is, however, a time- and resource-demanding process. As updates on validation guidance are conducted, the valuable components must be kept: Reliable data are and will remain fundamental. In 2016, the scientific community was made aware of the general crisis in scientific reproducibility—validated methods must not fall into this. In this commentary, we emphasize the central importance of ring trials in the validation of experimental methods. Ring trials are sometimes considered to be a major hold-up with little value added to the validation. Here, we clarify that ring trials are indispensable to demonstrate the robustness and reproducibility of a new method. Further, that methods do fail in method transfer and ring trials due to different stumbling blocks, but these provide learnings to ensure the robustness of new methods. At the same time, we identify what it would take to perform ring trials more efficiently, and how ring trials fit into the much-needed update to the guidance on the validation of NAMs.

**Supplementary Information:**

The online version contains supplementary material available at 10.1007/s00204-024-03736-z.

## Introduction: The practice of new method validation and the need to update

The development of the existing OECD Guidance Document on the validation and international acceptance of new or updated test methods for hazard assessment began in 1998. Intended to support confidence in test methods developed for regulatory applications, it culminated in the consensually agreed core OECD Guidance Document No. 34 on the validation and international acceptance of new or updated test methods for hazard assessment (OECD 2005 GD34). It provides a synopsis of the state of test method validation, in what is a rapidly changing and evolving area and today many/most novel test methods under development are, so called, New Approach Methodologies (NAMs). GD34 applies to experimental test methods in general, but most of the new methods are NAMs. There are several definitions of NAMs (ECHA 2016; OECD 2022, No. 356; Schmeisser et al. 2023a), all of which include in vitro methods, but may also refer to in vivo methods. This paper mainly refers to in vitro methods; but we should keep in mind that reproducibility, ring trials and validation are relevant to all methods including in vivo methods.

**Fig. 1:**
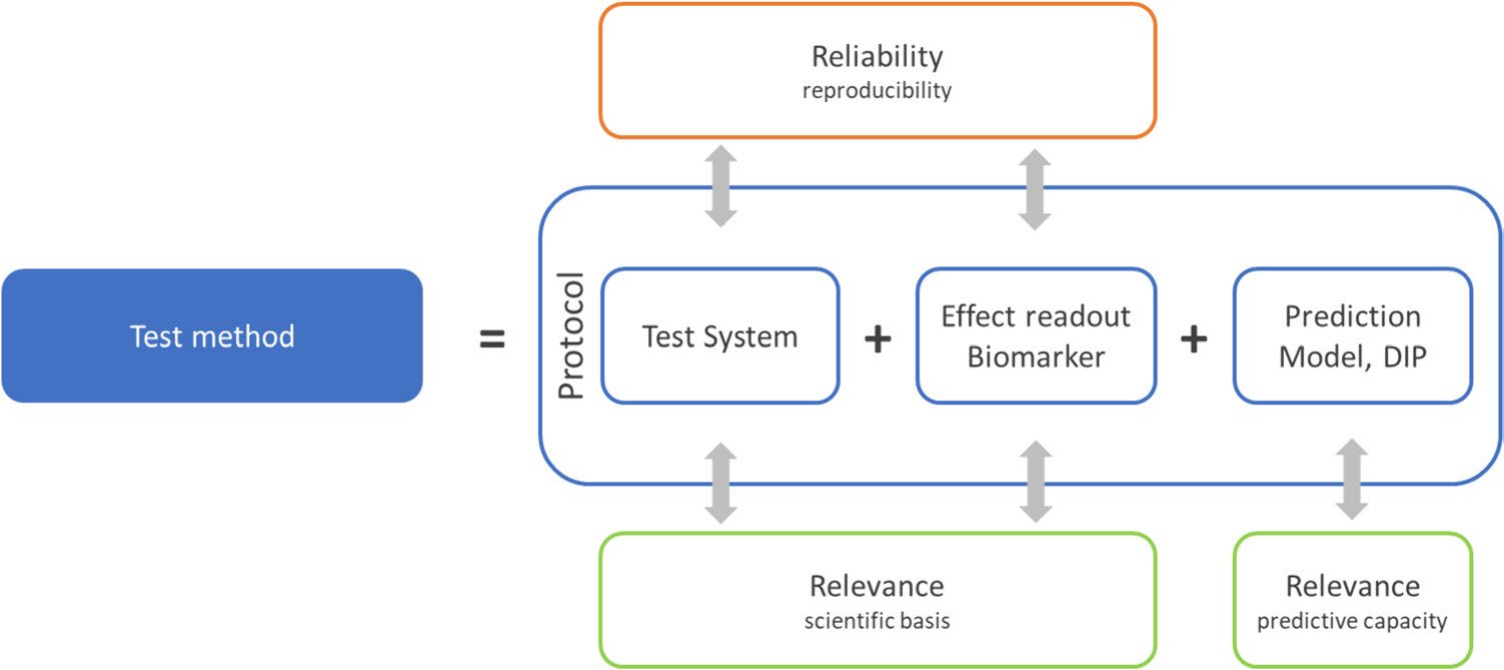
A schematic representation of a test method, its components, and its performance properties. (redrawn and modified from Worth and Balls 2001)

**Fig. 2:**
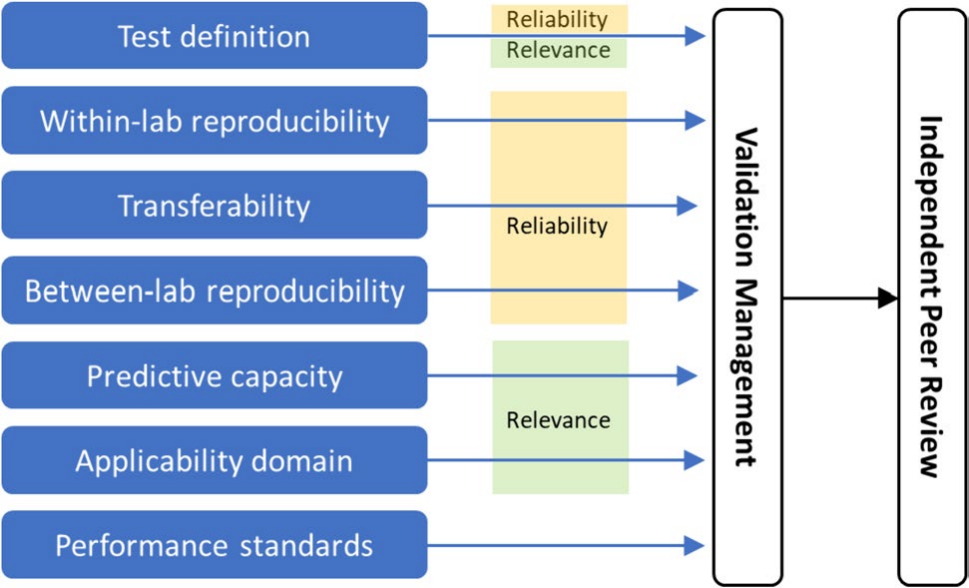
Modular approach of the validation process. (Redrawn from Hartung et al. 2004)

What a “method” is, its components and how to describe it, has been illustrated by the OECD (OECD 2017) others (e.g., Leist and Hengstler 2018). The OECD’s guidance document on “Good in vitro method practices” (GIVIMP) (OECD 2018, GD no. 286) describes the good practices for state-of-the-art in vitro methods applied to regulatory human safety assessment. It addresses elements of a test method such as materials and reagents, test systems and standard operating procedures (SOPs). Recently, Cöllen and co-workers summarized all the key test method elements to be: The purpose, the test system, the test chemical exposure scheme, and the endpoint (Cöllen et al. 2024*, *Fig. 1). The reliability of the test system is one of the aspects of a formal validation described in GD34 (OECD 2005) and comprises; within-laboratory and between-laboratory reproducibility (WLR and BLR, respectively), see Fig. 2.

The formal validation as described by this OECD GD is the prerequisite for a new method to become an OECD Test Guideline (TG) with application for regulatory purposes, particularly in keeping with the Mutual Acceptance of Data principle (MAD). In addition to reliability, the other main aspect is relevance which describes the extent to which the test method measures or predicts the (biological) effect of interest (OECD 2005).

With the ongoing transition from chemical hazard and risk assessment based on animal studies towards assessment relying mostly on non-animal data, the implementation of a multitude of novel experimental methods within industry and contract research laboratories, is needed, as well as adaptation of regulatory requirements. Established processes to assess the relevance and reliability of experimental methods in (eco)toxicology to gain MAD across regions are time consuming and often rely heavily on comparison of novel methods with reference data, primarily from animal studies, but also from available human data and using weight of evidence of all available data (Kolle et al. 2019, Hoffmann et al. 2008). These aspects appear to present barriers to the implementation of novel methods, at the speed desired by society and some stakeholders (Bhuller et al. 2024).

However, we also face a crisis in scientific study reproducibility. A survey conducted by Nature and published in 2016, reported that of more than 1500 scientists, more than 70% had tried and failed to reproduce another scientist’s work, and more than half had failed to reproduce their own studies (Baker 2016). Chemistry and biology were the worst offenders—and it is these fields that we depend upon most in the OECD TG programme. In the same vein, protocols are rarely published fully, so that others are hindered from reproducing the work. A study by Errington et al. (2021) reported that of 193 experiments, no protocols were fully described, and there were many barriers to conduct experiment replications. These concerns are being recognized, with efforts being made to promote reusable and open methods and protocols (e.g., Leite et al. 2023), and some journals have started accepting protocols as submissions.

Whilst the vision of replacing animal testing is of course a shared goal, the necessity to derive sound regulatory decisions to protect human health and the environment is of primary importance. The confidence of different stakeholders of chemicals management in NAMs will depend upon confidence in the reliability and relevance of the result delivered by the NAMs. Relevance of methods differs greatly for different settings. For example, in academia research, a method may be relevant when it can be used to exclude a specific key event. In the context of this manuscript, a relevant method is one which informs specific aspects of hazard or risk assessment, be that in a regulatory context or in industry risk governance. This requires that the test method addresses a relevant endpoint for chemical hazard and risk assessment, but also that the test method prediction model employed can demonstrate endpoint relevance.

The scope of this commentary is upon the reliability requirement, and we emphasize the central importance of transferability and inter-laboratory reproducibility assessment in the validation of experimental test methods, for chemical safety decision making and international regulatory purposes. We specifically critically address some commentary’s that are being put forward (e.g., Chemwatch 2023, 2024) where ‘ideas being mooted’ include making ring-trails optional. We explain why this is simply not an option.

The validation data generated to develop a TG is considered to be the most rigorous, providing the greatest confidence, to ensure acceptability across many regulatory jurisdictions, as well as providing legal certainty.

We show that confidence in NAMs is a prerequisite for uptake of the methods into chemical and consumer goods industries environmental health and safety governance processes and decision-making, beyond compliance with the different regulations. (Note that our focus is not on company-internal application and validation of NAMs for R&D screening purposes.)

“There is no doubt that quality assurance of methods must be based on method definition (including purpose and applicability domain) and reproducibility. This is actually also the easy part; it gets difficult when scientific basis and relevance are addressed.“ (Hartung 2010). This insight remains true: Whilst assessing the relevance of new methods in regulatory toxicology is essential; independent assessment of a method’s reproducibility remains a fundamental and essential component to establish confidence in a test method.

With the increasing development and applications of NAMs for regulatory purposes in recent years, it has become timely to review and update the OECD GD 34 on validation for the OECD Test Guideline Programme (TGP) (OECD 2005), to improve guidance for future NAMs application for regulatory purposes, particularly in keeping with the MAD principle.

There are several conceptual views as to how to adapt validation to current needs (Lanzoni et al. 2019, van der Zalm et al. 2022, Marx-Stolting et al. 2023); but these largely address validation of the relevance of NAMs that highly depends upon the intended use. Here we make the case for ensuring the reliability of NAMs with ring trials. By drawing upon different learning experiences gained from ring trials we discuss how they should be performed efficiently to benefit the validation process, without unduly wasting valuable time and resources. Our goal is to avoid a reproducibility crisis in regulatory toxicology.

## Information box: plain language terminology

**Table:** 

Validation	The assessment of the reliability (or reproducibility) and relevance (predictive capacity) of a particular test, approach, method, or process with an associated prediction model established for a specific purpose ‘Prevalidation’ is used to describe the first stages of the validation process. Ideally the optimisation and characterisation of the test method has been completed and transferability is established and within lab variability is assessed
Devalidation	The removal of a validation or the failure to prove a method to be valid. (https://en.wiktionary.org/wiki/devalidation)
Standard prospective validation	The review and analysis whereby a method is demonstrated to do what it purports to do, as shown by the results generated in a ring trail of three or more laboratories. Focus herein is with respect to prospective validation
Retrospective validation	The review and analysis as to whether a method does what it purports to do based on accumulated historical results
Modular Approach to Validation	see Fig. 2
Ring trial	Also termed e.g., “inter laboratory comparison”, “ring-study” or “round-robin” An external reproducibility control in which a test manager distributes test items to the participating laboratories, to perform the same study according to the same protocol. If possible, it is statistically planned, and test items are blind-coded
Test definition	Defines the scientific purpose of the test (mechanistic basis and/or toxicological endpoint)
Within-laboratory variability	Also called intra-laboratory variability. Defines how well a test result is reproduced in the same lab using the same equipment based on repetitive testing
Transferability	Assesses the practicalities of the test and is essential for robustness. It provides information whether the protocol is sufficiently detailed and how much training may be necessary for the evaluation of the within- and between laboratory reproducibility
Between-laboratory variability	Also called inter-laboratory variability. Defines how well a test result is reproduced between usually at least three labs based on repetitive testing in a ring trial
Predictivity, Predictive capacity	Describes how well a test with a defined prediction model predicts a reference outcome (effects in humans and/or animals). The predictive capacity is usually described by parameters such as sensitivity (true-positive rate), specificity (true-negative rate) and overall and balanced accuracy
Applicability domain	The applicability domain of a particular test is defined for or can exclude chemical classes, product types and/or physiochemical properties
Performance standards	A set of reference chemicals usually defined after completion of validation. These chemicals can then be used to conduct a so called “me-too” validation of sufficiently similar methods
Reliability	Defined by the within- laboratory reproducibility (WLR) and between laboratory reproducibility (BLR)
Relevance	The relevance of a test method describes the relationship between the test and the effect in the target species and whether the test method is meaningful and useful for a defined purpose, with the limitations identified. In brief, it is the extent to which the test method correctly measures or predicts the (biological) effect of interest, as appropriate. Regulatory need, usefulness and limitations of the test method are aspects of its relevance
Adoption as OECD (Test) Guideline (TG)	When successfully validated and following independent peer review, a test method can be proposed as a Test Guideline, which, if consensually approved by the Working Party of the National Coordinators of the Test Guideline Programme at OECD (WNT) can be used by all OECD member countries under the Mutual Acceptance of Data (MAD) agreement
Test Guideline revision	OECD TGs are updated (revised) to reflect the state-of-the-art. A member country submits a standard project submission form that has to be approved by the WNT of the Test Guideline Programme at OECD for such an update (or revision) to happen. Revisions or updates also include the addition of methods to existing test guidelines, e.g., after a so-called performance standard based ‘me-too’ validation
TG augmentation	e.g., the addition of extra endpoints and or expansion of the chemical applicability domain of a TG
Me-too test method	Performance Standards for TGs are based on a scientifically valid and accepted test method, that can be used to evaluate the reliability and relevance of other analogous test methods (colloquially referred to as “me-too” test methods) that are based on similar scientific principles and measure or predict the same biological or toxic effect (OECD 2005)
Integrated Approaches to Testing and Assessment (IATA)	IATA combine multiple sources of information to conclude on the toxicity of chemicals. IATAs may include existing information from the scientific literature or other resources, along with newly generated data resulting from new or traditional toxicity testing methods to fill data gaps. These approaches are developed to address a specific regulatory scenario or decision context. IATA comprises ‘intelligent’ or integrated testing strategy (ITS) and Defined Approaches (DA). (OECD 2016a, b*, publication no. 265*). The term ‘IATA’ is still frequently used synonymously with ‘integrated testing strategy’ (ITS), and both these terms are sometimes used synonymously with ‘weight-of-evidence’ (Sauer et al. 2016)

*Please see OECD guidance document no. 34 (OECD 2005) for formal definitions.

## The current state of validation

### What is ‘validation’ for regulatory purposes? Why is it important?

Validation is a scientifically anchored process that serves to demonstrate the reliability and relevance of a method for a particular purpose, for example, hazard classification or safety assessment of uses of chemicals (Bruner et al. 1996, Bas et al. 2021, Holzer et al. 2023a, 2023b). Validation is an essential requirement for TGs such that they can be used under MAD in all OECD member countries (as well as non-member provisional and full adherents), and thus meet legal obligations for all stakeholders. TGs are primarily intended for hazard identification and characterisation purposes, for application in risk assessment in various formats depending upon chemical sector and regulatory jurisdiction.

**Fig. 3:**
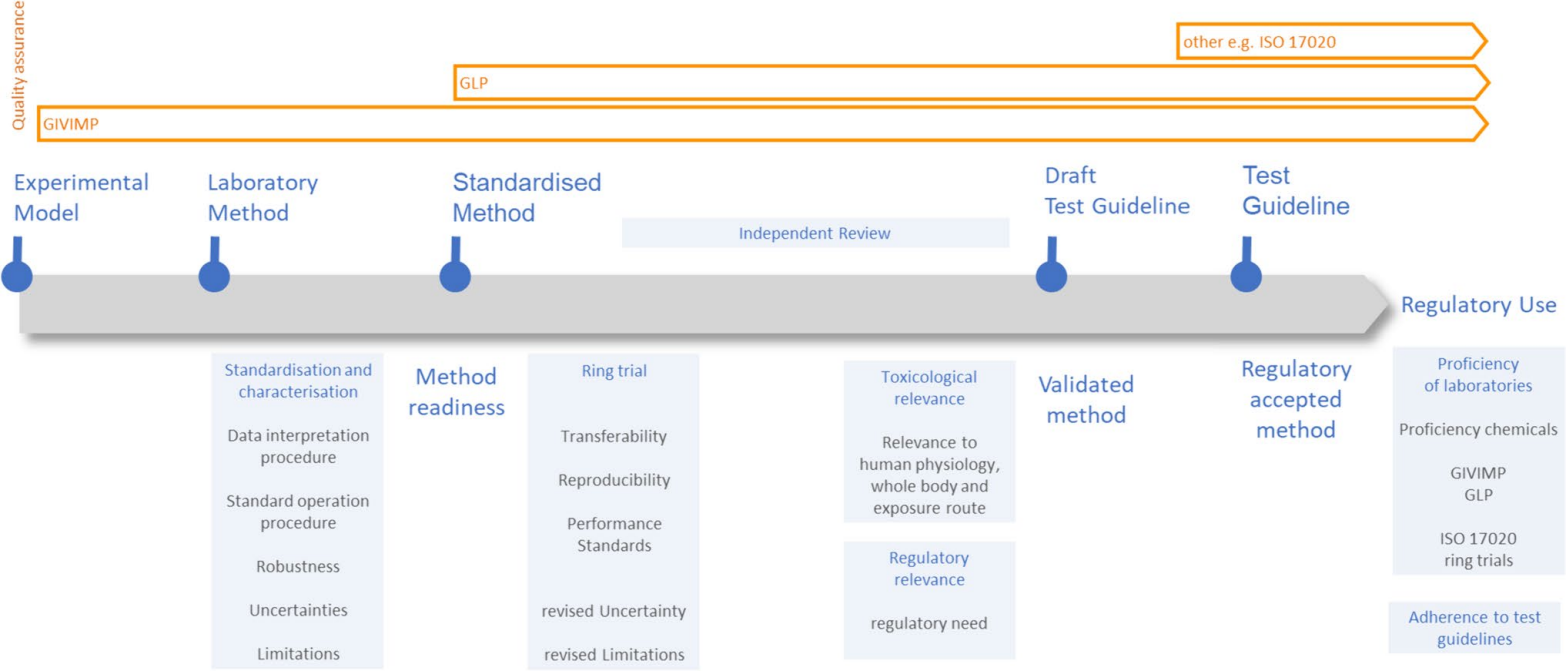
Steps from method development to method validation and finally to its regulatory use. Some aspects, like the toxicological and regulatory relevance, are considered at the first point of assessing a new method’s readiness to go into validation

Validation work of in vitro methods/new approach methodologies is/are usually organised in modules, guided by the modular approach (Hartung et al. 2004), Fig. 1. Early modules are aimed at biological relevance, test method optimisation, detailed documentation, reproducibility within a laboratory and transferability. Later modules address between laboratory reproducibility, predictivity and applicability domain. Full validation usually includes ring trials with blind-coded test substances, which provides unbiased evidence for BLR and predictivity assessment. The validation module work serves the purpose of providing unbiased and conclusive evidence for “trust-building” between the parties involved: principally the scientific and regulatory community, including industry risk assessors and managers. This additionally ensures legal certainty. See Fig. 3.

While understanding that data are related to their relevance, trust is related to reliability, and this builds confidence. Well described, and well characterized candidate test methods facilitate subsequent more rapid validation. The more test method developers engage in this process early on, the greater the increase in the efficiency of the ring trials and the prospect of a successful validation will be. This should be the reason enough to raise the awareness for the need of good in vitro method practices (GIVIMP), high levels of standardisation and scrupulous method description during the method development stage by test method developers, including academic researchers.

### The Story behind the proposed update to Guidance Document no. 34

OECD TGs are used e.g., by governments, industry, and independent laboratories to assess hazards and safety of chemicals. The use of TGs that are based on validated test methods promotes the generation of dependable data for human and animal health and environmental hazard assessment (OECD 2005). TGs fall under the MAD agreement (OECD 1981), and this is a foundation of the OECD TGP. The MAD framework ensures the generation of high quality and reliable non-clinical test data for regulatory purposes. Good laboratory practice (GLP) provides the quality standards for experimental testing and TGs provide the scientific standard. GLP was implemented in the 1970’s in response to fraudulent data submitted to regulators. Regulatory authorities receiving the data under the MAD agreement know that particular quality and scientific standards were followed and that they do not have to re-evaluate the concomitant test protocol to determine its robustness, as it has consensus by countries via the OECD TGP. The OECD Council Act on MAD (OECD 1981) states that “Data generated in the testing of chemicals in an OECD Member country in accordance with OECD Test Guidelines and OECD Principles of Good Laboratory Practice shall be accepted in other Member countries for purposes of assessment and other uses relating to the protection of man and the environment.”

While regulatory data requirements are government prerogatives, as are the interpretation of test results, importantly, under MAD, no repeat testing is needed for the same data requirement. However, “acceptance” does not automatically mean “use” of data. The more compatible data requirements are between countries, the more beneficial MAD will be globally.

Building upon the MAD principle, OECD member countries developed a guidance document on “Validation for in vitro and in vivo Test Guidelines”, to provide the ‘general principles, important considerations, illustrative examples, potential challenges and the results of experience gained in the area of test method validation’. This was published in 2005 and at that time most TGs were still describing in vivo methods, but it was acknowledged that an increasing number of test methods coming forward were likely to be in vitro. In the intervening years the TG landscape has and continues to undergo evolution, more and more in vitro test methods are coming forward for validation and TG development, together with a great deal of discussion regarding how to optimally combine them for a given hazard as part of Integrated Approaches for Testing and Assessment (IATA). This is because, overall, they addressed Molecular Initiating Events (MIEs), and Key Events (KEs) of adverse outcome pathways (AOPs), and modes of action, leading towards an adverse outcome, but taken on their own, are insufficient to characterize a hazard to make a regulatory decision, in the way in vivo studies were and are assumed to do. Results from different NAMs can, therefore, be integrated in an IATA and assessed in a weight of evidence approach. Some IATAs are indeed built on KEs of an AOP (OECD 2020, no. 329); if an IATA or part thereof becomes prescribed and more structured, and validated, such that it is a defined approach (DA), it can become a TG under MAD.

As an indication of the scale of the shift towards in vitro TGs, while the projects on the current OECD TGP workplan in Sect. "The current state of validation" for biotic systems (environmental) are still mainly in vivo (e.g., fish, avian, invertebrates), the majority of more than thirty projects in Sect. "The way forward", on human health, are NAMs related (OECD 2023d). As a consequence of the greatly increasing momentum in the development and validation of in vitro test methods, sometimes in combination with in silico tools (e.g., OECD 2023c, Test no. 442E, OECD 2023a, Guideline no. 497), the WNT agreed to revisit GD 34 and update the guidance, in line with this evolution. The project entered the OECD TGP workplan in April 2023, and work has been initiated. A major underlying concern behind the proposed update to GD 34 is the length of time it takes to successfully validate and achieve TG adoption.

The timelines for validation, peer review and the adoption process can vary greatly for several reasons, but often the holdup is right at the start, if e.g., the test method has not been sufficiently optimised, before laboratory transfer. Examples of validation exercises that were rapid, include the kDPRA which took only three years including ring trial and formal adoption process (OECD 2023b, Test No. 442C), the KeratinoSens™ which took less than a year for the ring trial (mid 2009 to early 2010) and was adopted by the OECD in 2015 (OECD 2022b, Test No. 442D) and the fish gill cell assay which took less than two years (from 2015 to 2016) for the experimental phase but was adopted five years later by the OECD in 2021 (OECD 2021a, b, Test No. 249). Some examples that have taken longer, together with the reasons why, are included in Table 1.

**Table 1:** Some examples of stumbling blocks during ring trials for the validation of test methods

Addressed MIE, KE or AO	Test method	Phase in which the problem occurred	Challenge encountered	Action taken and whether successful	References
Estrogen Receptor (ER)	ER TA TG455	BLT	First ER CALUX construct (*Legler *et al*. 2002*). Failed transfer to other labs during, although WLR in lead laboratory first appeared to be encouraging	Novel clone of U2-OS line stably co-transfected with human ERα (pSG5-neo-hERα) and a pGL3 -based reporter construct containing 3 EREs (pGL3-3xEREtataLuc) (*Sonneveld *et al*. 2005*). Improved stability and luciferase levels and was successfully transferred (*van der Burg *et al*. 2010*) validated and adopted (OECD TG 455)	van der Burg et al. (2010), Sonneveld et al. (2005)
Androgen Receptor (AR) transactivation assay (TA)	AR TA TG457	Excessive variability, resulting in poor WLR	GR cross talk in cell line, first results from the lead laboratory indicated confounding of AR responses	New cell line/construct developed, with GR knockout cell line. New cell line characterized. Successful validation meeting Performance standards for TG 457 following construction of GR knockout cell line	Park et al. (2021)
Estrogenic responses	MCF7 proliferation assay	WLT BLR?	US ICCVAM Poor performance of antagonists BLR was 54% (14/26)	Not continued post 2012	https://ntp.niehs.nih.gov/sites/default/files/iccvam/methods/endocrine/mcf7/mcf7-valstudyreport-19jun12-wcv2-draft.pdf
Skin Sensitization	h-CLAT	BLR	BLR below target. A number of RT conducted (2 phases or 1RT, and then 3RT) to address issues with BLR. Protocols adapted based on ring trial experiences	Approved	Sakaguchi et al. (2006, 2010)
Skin Sensitization	Myeloid U937 Skin Sensitization Test	BLT	Problems encountered in BLT	Stopped, restarted and finally approved with new protocol (U-Sens)	https://tsar.jrc.ec.europa.eu/test-method/tm2009-05
Skin Sensitization	NCTC2488 IL-18 assay	BLT	Poor reproducibility in pre-validation	Stopped after pre-validation study	Teunis et al. (2013, 2014) unpublished and Robert Landsiedel personal communication
Skin Sensitization	IL-18 epidermal equivalent assay	BLT	low sensitivity and problems with meeting the acceptance criteria	Stopped after pre-submission to ECVAM Validation study not reported	https://tsar.jrc.ec.europa.eu/test-method/tm2012-05
Embryotoxicity	Embryonic Stem cell test (human and murine)	BLT	Pre-validation transferability Issues with bacteriology dishes to allow embryoid bodies to be maintained in suspension culture. Despite adherence to supplier and order number, high rate of failures due to an unacceptable rate of adhesion of the embryoid bodies	Upon receiving a shipment of the German vendor-sourced bacteriology dishes, laboratories were not able to demonstrate proficiency in the test method. Probable reason: Different sterilization/irradiation technique for the same type of dishes for the USA market This multi-laboratory pre-validation revealed the importance of evaluating the test methodology in different geographical locations regardless of the laboratories’ technical competence Not progressed in validation bodies (EURL ECVAM) as yet	ESNATS 2008–2013 Embryonic Stem cell-based Novel Alternative Testing Strategies | ESNATS | Project | Fact sheet | FP7 | CORDIS | European Commission (europa.eu) Scholz et al. (1999)*, *Raabe et al. (2009)
Cytotoxicity assay	3T3 NRU	BLT	The laboratory experienced with working with 3T3 cells in these cytotoxicity assays generated IC50 data for the NHEK cells that were substantially statistically different for the positive control relative to that of the lead laboratory and the naïve laboratory	A face-to-face laboratory training and protocol review identified that the experienced laboratory had adapted the SOP and was adding a culture-enhancing treatment to their culture plates for the NHEK cells, thereby reducing the cells’ sensitivity to the positive control As the transferability and reliability activities in Phase 1 were conducted in several laboratories, rather than just one, the ability to detect such unwanted SOP deviations in following the protocol specifications was identified	https://ntp.niehs.nih.gov/sites/default/files/iccvam/docs/acutetox_docs/brd_tmer/brdvol1_nov2006.pdf
Skin irritation Test (SIT)	Performance Standards-based “Me too” validation of the SIT using the EST-1000 (now EpiCS) tissue model	WLR	Cross Atlantic validation study. The laboratories were fully proficient in the test method due to prior relevant validation experience However, on several occasions, transatlantic shipments of the EST-1000 tissue models were delayed by at least one day. Without guidance to the contrary, validation testing using these tissues was conducted However, WLR was adversely impacted due to correlation between invalid test runs and non-concordant results with delays in tissue deliveries	This multi-laboratory validation revealed the importance of evaluating the test methodology in different geographical locations regardless of the laboratories’ technical competence	Hans Raabe, personal communication
Genotoxicity	Comet in vivo	BLR	In three ring trials with coded substances various issues were discovered. In the phase 2 of the prevalidation, the positive control was negative in 2 labs and the predictions (negative vs positive) were inconsistent across 5 labs. In the next phase, issues were reduced to an extent to move to full validation. In the first validation phase, 4 substances were tested in a ring trial. The only new substance 2-AAF, which requires metabolic activation, was not reproducible	Iterative improvement of the protocol. The issue with 2-AAF, triggered an expert review of other available data (https://web-archive.oecd.org/2013-02-07/223021-Intra%20and%20inter-lab%20reproducibility%20invivo%20Come.pdf)	Uno et al. (2015a, b)

It is recognized that for some stakeholders, the length of time it can take to develop and adopt TGs is disappointing, and shortcuts are being proposed to speed up the adoption process. Principal amongst these proposals are suggestions to make the process ‘lighter’ by for example skipping multi laboratory ring trials. However, the examples provided in this commentary demonstrate that multiple other factors than the time taken to conduct ring trials themselves, should equally be assessed for their contribution to time until adoption. Sect. "Stumbling blocks in method optimisation and for reliability: What can go wrong and why?" describes stumbling blocks encountered during ring trials, on the journey towards becoming an OECD TG.

### Funding of validation activity

Early on, the European Commission fully funded validation projects of new methods addressing human health hazards, e.g., for skin corrosion and irritation, embryotoxicity, and cell transformation assays (Fentem et al. 1998; Fentem et al. 2001*; *Spielmann et al. 2007; Genschow et al. 2002*; *Corvi et al. 2012). With full funding, it was possible to optimally adhere to validation principles, such as independence, minimisation of biases (e.g., chemical selection, coding) and sound experimental designs. Later, validation projects were co-financed by public and private sponsors, e.g., h-CLAT, DPRA and U-SENS. This remains a viable option (PSCI et al. 2022) and is the model followed in the recently established French (pre-)validation platform for endocrine disruptors, PEPPER (L'association Pepper (ed-pepper.eu)). In these, the public contributor can maintain the necessary independence. In addition, (pre)validation activities were included in large European Commission funded research projects, e.g., AcuTox (Clemedson et al. 2007), ReProTect (Hareng et al. 2005), ESNATs (Bolt 2013; Krug et al. 2013) to GOLIATH (Legler et al. 2020). It needs to be acknowledged that, while such large publicly funded projects can advance the assessment of NAMs, unfortunately, they are less suitable to validate NAMs formally and fully to completion/adoption, due to a lack of expertise, focus, allocated time and dedicated funding. Centrally managed validation activities may also be an option, such as that conducted by the EU-NETVAL activity on in vitro methods for the identification of modulators of thyroid hormone signalling (EC-JRC 2023), but it is notable that the European laboratories have been self-funding in this preliminary exercise, with other platforms taking forward further validation funding, as seen for example with the PEPPER platform. Finally, more and more primarily privately sponsored validations are being conducted, e.g., RSMS/RSCOMET (*Reisinger *et al*.; Pfuhler *et al*.*), and SkinEthic HCE Time-to-Toxicity test method (Alépée et al. 2022) and Sens-IS (Cottrez et al. 2016), GARDskin® (Johansson et al. 2019), LuSens (Ramirez et al. 2016) and kDPRA (Natsch et al. 2020; Wareing et al. 2020), for these, the avoidance of conflicts of interest are a main challenge.

In the US, validation has generally been government funded, similarly for Japan. Validation exercises are often international collaborations, with each partner resourcing the participant laboratories according to the resources that they can leverage, in their sector or country.

## Stumbling blocks in method optimisation and for reliability: What can go wrong and why?

In validation, issues often arise both when transferring a test method and when subsequently assessing the reproducibility of results, across laboratories in a ring trial. It is important to understand that these two modules (Fig. 2) are tightly linked. In our experience, the preparation of a blind-coded ring trial, and the definition and review by test laboratories of the Standard Operating Procedure (SOP) in the transfer phase, needs to be conducted in a very thorough manner, because in the blind-coded phase no further adjustments are possible. As an analogy, when one is preparing for a long bicycle road race, or a long tour, one would want to ensure that you have the optimum bicycle, and that it is thoroughly serviced, as it is unlikely that there will be a service technician along the road.

At the core of a method, validation assesses robustness and repeatability. During a ring trial, obstacles and shortcomings are detected, and the process offers the opportunity to improve the method—or devalidate it.

As an illustration, PEPPER have already observed such issues and have published a list of “… aspects that are not directly related to the method’s repeatability, reproducibility …”, among them: Imprecision and different interpretations of the SOP and errors in reporting data (Rivero Arze et al. 2023). There are more examples when looking at the ring trials conducted during the last 20 years. Table 1 provides illustrations of problems encountered whilst validating test methods and running ring trials.

As can be seen, a variety of problems are uncovered in ring trials—demonstrating that ring trials are an effective and essential tool in assessing a new method. The problems that arose were not only due to lack of protocol details, or lack of adherence to the protocols, but for example were also due to differences in plastic ware sourced in different regions—even if produced by the same company. In some instances the test methods were simply not sufficiently optimized before entering validation, leading to cell line and receptor construct failure on transfer, or if lacking in test system characterisation, inadequate analysis of technical issues. Sometimes the problem was logistical, with shipment delays, and learning that these were critical for the sensitive performance of the test method.

## The way forward

### Lessons learned: avoiding failures right from the start of method development

With increasing concern regarding differing laboratory cell culture practises, the need to improve guidance for test method developers to address many of the areas of weakness observed in optimisation and early validation stages of test methods was recognised, and a comprehensive guidance document on good in vitro methods practices was published in 2018 (OECD 2018 GIVIMP).

This guidance is a comprehensive quality assessment framework in many aspects related to in vitro methods, and it goes beyond other quality standards such as good laboratory and cell culture practices. It provides a set of quality standards to improve both the quality of and confidence in newly developed, as well as routinely executed in vitro methods. The guidance is addressed to test method developers but also to down-stream users. Demonstrating adherence to GIVIMP, builds stakeholder confidence in the method developer, the method itself, and the laboratories conducting the method. In the context of past validation studies, following the principles of GIVIMP, in particular, with respect to the test system characterization (which includes identity and contamination checks and sufficiently detailed documentation), would have circumvented many of problems listed in the table above. The OECD workshop report with respect to needs for human serum use (Jacobs et al. 2019, 2023) provides additional supplementary information for the GIVIMP in relation to reporting on human serum use and is intended as a checklist for test method developers to ask of their suppliers, to stimulate better practise.

### Transition from qualitative validation to quantitative validation

While we strongly advocate to keep ring trials as an essential part of method validation, the way ring trials are designed and evaluated certainly should evolve. NAMs developed and validated over the last two decades especially in the field of skin and eye irritation, and skin sensitization were often developed to classify chemicals into “positives” and “negatives”, i.e., an answer which can directly be used in chemical hazard assessment for classification and labelling. This has also been reflected in how in vitro test method ring trials were conducted in the recent past, as prioritisation for subsequent in vivo testing was also a primary objective in many cases, such that the evaluation of ring trials results was often largely based on how well a method could allocate chemicals into positives or negatives. With the evolution of IATAs, for the NAMs developed more recently where good concentration response data are generated, this is now also the focus.

Going forward, test development needs to identify key biological events or pathways relevant for the endpoint of interest and also develop quantitatively informative tests to address this event with e.g., an in vitro method. These tests will need to provide continuous data, e.g., concentration–response information, or kinetic information, which can be summarized in key parameters such as e.g., a range of effect concentration (EC) values, metabolic rates, etc. Evaluation of intra- (WLR) and inter-laboratory (BLR) tests will then need to address variability of these parameters, i.e., consider the full information content of the test. This quantitative approach will influence how ring trials are being set up: In the case of the tests for skin and eye irritation, the validation studies often included a larger group of chemicals, sometimes up to 24 per laboratory. Indeed (due to the low information content of a yes/no answer), significant numbers of chemicals are needed to answer the question: Will a laboratory reach 85% intra- and 80% inter-laboratory reproducibility of allocating chemicals into two groups across a predefined threshold? Chances of success of such validations have also been influenced by the number of chemicals in the test set with an intrinsic activity close to the decision threshold. On the other hand, comparing key parameters of the continuous data will often give a good indication of the intrinsic variability of the test (biological and experimental variability within a laboratory) and about the effect of different operators in different laboratories (robustness of the SOP and the experimental procedure across laboratories). Dependent on the method at hand, a scientifically justified number of chemicals should be used, enabling the characterisation of the influence of physico-chemical properties on variability across the concentration response spectrum. Pre-validation work on the chemical applicability domain and predictive capacity should inform selection of the number and type of chemicals to be included in ring trials.

As a point of reference, the ring trials lately conducted in the environmental toxicity field may serve here as guiding posts: In the RT-gill W1 assay leading to OECD TG 249, EC_50_ values for cell viability were measured for six chemicals tested in five laboratories. In the trout liver metabolism assays leading to TG 319A and 319B, five chemicals and a positive control were tested in six laboratories. In both cases, each chemical was tested three times for intra-laboratory reproducibility in each laboratory and in both cases continuous values (EC50 or metabolic rates) were reported and assessed, and not only yes/no answers.

Other good examples include the Androgen Receptor Transactivation Assay CALUX test method in TG 458, where the data quality and number of chemicals tested were comprehensive, the use of the concentration–response data could have been maximized (ESAC 2020). However for TG 458, the older dichotomous prediction model was followed to create one performance-based TG for three different assays (OECD 2020; Milcamps et al. 2021; Park et al. 2021). This was perhaps a lost opportunity, but one that could still be revisited (Jacobs et al. 2022a, b).

With this move towards quantitative methods, it may make sense to come up with standardized statistical parameters to compare reproducibility of dose/concentration–response or other continuous information during the validation process. Such uniform measure of quantitative variability of continuous data could be applied to past and future validations to yield a benchmarking of reproducibility for different biological tests with continuous information.

One of the most important benefits of quantitative validation is that it is forward looking—if new decision thresholds are being introduced, as the case in TG 497 (Defined Approaches on Skin Sensitization), the quantitative validation data may be consulted to evaluate robustness of the new prediction model, and one doesn’t need to restart the validation process all over again. It is such considerations as these, on evolving ring trials that need to be part of the revision of GD34.

### What is a “validated method”—a changed mind-set

The original validation exercises for skin and eye endpoints covered all aspects of the modular approach (see Fig. 2) including reliability and predictive capacity. However a validation study focused on reliability only, is likely to be a more frequent case in the future. The decision as to biological/scientific relevance should be taken first, before determining reliability through ring-testing. This needs to be understood by both toxicologists and regulators—reducing any potential confusion as much as possible. Some of the first mechanistic tests that were validated only for reliability and initially not directly for the prediction of in vivo outcomes, are the different test methods on endocrine activity. These tests indicate the potential (and potency) of a chemical to interfere with endocrine pathways *in vitro* and the validation focused on the reliability (i.e., reproducibility) question. Following the ‘classical’ approach—the ‘prediction model’, rated chemicals as “positives” or “negatives” although for TG455, the HeLa Oestrogen receptor SOP developed by Japan—the first endocrine activity adopted TG, a weak positive could be identified with a PC10 (the concentration inducing a 10% response in relation to the positive control). More recently, Weber and coworkers suggested a prediction model for the inhibition of the deiodinase enzyme 1 (DIO1, is deiodinising thyroid hormones, *TSAR, Test method number TM2019-10*) based on full or partial inhibition and the potency compared to a well-described inhibitor with adverse effects in humans (Weber et al. 2022; Weber et al. 2023).

For mechanistic assays, binary hazard prediction models should not be the only prerequisite for OECD adoption: Mechanistic biological tests validated for reliability only, should be clearly understood as such, and if at all possible, on the basis of the data generated, not include only ratings such as “yes” or “no”, but also address potency—if the quality of the data are sufficient to do so. Then follow-up work can address how the quantitative data can be optimally integrated. Here there is a key role for in vitro studies in relation or physiologically based toxicokinetic modelling and the evaluation of a potential internal dose (OECD 2021a, b), i.e., in vivo to in vitro extrapolation (IVIVE) e.g., as with a suitable IVIVE model, or in an IATA, to translate the in vitro biological activity into a prediction of an apical endpoint. Whilst the demarcation of ‘positive’ or ‘negative’ are useful for classification and labelling purposes (Jacobs et al. 2022a, b), as we move towards test methods that also will provide quantitative mechanistic in vitro assay outcomes it will be pertinent for the update to GD34 to develop clear guidance for both approaches.

### Practical implications

Advances in synthetic biology, chemistry and material engineering processes are leading to the manufacture of new substances or their application in novel ways, impacting a multitude of industrial, consumer and pharmaceutical sectors. Whilst these innovations are high-growth commercial opportunities, their regulatory safety assessment is challenging as current TGs for hazard assessment are seldom compatible with emerging technologies, having been developed for chemicals but not different forms, such as nanomaterials. Ambiguous, unreliable data can stall or even prevent development of new products, whilst reducing environmental and human health protection.

As OECD GD 34 (2005) has entered a period of revision and update to align with these developments and facilitate their sustainability and safety, the following aspects are of primary concern.

1. That validation across several laboratories is key for global acceptance (e.g., legal certainty), and experienced validation management increases the likelihood of success.
2. That the exercise needs to be inclusive for all key stakeholders.
3. That the MAD principle needs to be protected. Any damage to the MAD principle will impact negatively upon the global chemical industry and public and environmental health alike. We will go backwards, and if this results in the reduction of chemical testing harmonisation globally, it will lead to a great deal more repeat testing in both in vitro and animal models, negating our advances in the 3Rs and improved harmonisation over the last 30 years, sending us back to the 1950’s.

Modernisation of GD 34 is needed, fully embracing these three core pillars. Optimisation and ensuring the reliability of NAMs for regulatory purposes will improve future regulatory practice and guidance and facilitate innovators to bring robust cutting-edge technologies to market. Therefore, supporting guidance and training is also needed for the identification and recruitment of suitably experienced laboratories, together with independent chemical selection appropriate for the chemical regulatory applicability domain that the test method is intended to address, blind coding, logistics and biostatistics.

Finally, and fundamental to the ring trial validation work, is the provision of adequate, dedicated, and stable long-term funding.

The costs for ring trials should be calculated early on, communicated, and should be included in planning of further steps after the initial method development. There can be different funding models: For those laboratories who participate in ring trials to adopt the method for commercial use, ring trials may be part of the business case and could be self-funded, or external funding could be sourced from science-to-business programmes. Academic laboratories will, however, need external funding.

With increasing data requirements, both regulators and industry must be confident that different laboratories can perform an assay with coherent and reproducible results. A lack of confidence because of limited reliability, will hinder both regulators and industry. This can delay legal decisions based on the results of NAMs, as they are likely to/may be challenged, on the basis of the robustness of the NAMs. A lack of confidence will also hinder industry, due to environmental health and safety governance, in placing chemicals and products on the market, particularly in the innovation space (which is crucial for the aim of augmenting the sustainable uses of chemicals), and for both, a continuation of animal testing for product safety decisions becomes more likely, to be confident of negatives or positives. Regulators need to make strategic decisions based upon a confident understanding of the data and its quality. Only then can they, as well as industry, take a proportionate and defensible approach towards the identified hazard and risks. A lack of confidence here will, therefore, slow down the uptake of NAMs into legislation, across the globe. Thorough pre-validation work (such as a robust protocol with acceptance criteria and data interpretation procedures, i.e., defined as an SOP, proof of intra-laboratory reproducibility as well as clear understanding of any intellectual property right issues) at the outset will facilitate more rapid validation in particular for the inclusion of NAMs and ensure that discussion regarding legal certainty is minimized. Ensuring that the method is sufficiently mature, relevant and addresses a key information gap as well as having support from the regulatory and industry stakeholders, will speed up the validation process.

## Key messages

1. Validated methods are essential to generate reliable data to ensure safe handling and safe-by-design chemicals. Chemical industry and authorities regulating these depend on reproducible and relevant (eco)toxicological data to fulfil legal requirements.
2. Ensuring the reproducibility of a laboratory protocol and assessing the relevance of the data obtained with this protocol for hazard and risk assessment could potentially be separated. The scientific/biological relevance assessment needs to be revised to fit modern (eco)toxicology; whereas the reproducibility is and remains a fundamental basis for confidence in the quality of the data.
3. Reproducibility requires a robust, standardised, and well-described laboratory protocol which is transferable to other laboratories. This must be proven via ring trials.
4. New laboratory methods have failed in ring trials for different reasons. The success and efficiency of ring trials and the entire validation process can be increased with thorough preparation and effective conduct of ring trials.
  - Thorough preparation needs to follow GIVIMP, at the outset of test method development, to develop an accurate and comprehensive SOP, with careful protocol transfer to other laboratories (prevalidation).
  - Effective conduct of ring trials requires dedicated and capable laboratories, knowledgeable planning by experienced and committed management, together with reasonable funding.
  - In addition to information on the reproducibility and accuracy of a method, ring trials should also generate and report continuous data, as it is core to the development of IATAs and advanced DAs.
5. There is a need to establish training and mentoring programmes in the validation of NAMs, and the design and evaluation of IATAs, for both test method developers and regulators.
6. Adequate, sustained, and substantial funding for both validation (including management and statistical evaluation) and training is urgently needed.

With good preparation and professional, dedicated conduct, ring trials are neither an undue hurdle nor is the laboratory testing of a ring trial a major hold-up in a validation processes, but rather the touchstone of a method’s reproducibility and consequently also an important part in providing legal certainty for the method.

## Disclaimer

This opinion piece represents the view of the authors and not necessarily their organisations.

### Supplementary Information

Below is the link to the electronic supplementary material.

Supplementary file1 (DOCX 20 KB)

## Abbreviations

2-AAF: 2-Acetylaminofluorene
AI: Artificial Intelligence
AO: Adverse Outcome
AOP: Adverse Outcome Pathway
BLR: Between Laboratory Reproducibility
BLT: Between Laboratory Transfer
DA: Defined Approach
CRO: Contract Research Organisation
DASS: DA for Skin Sensitisation
DIO: Deiodinase enzyme
EC: Effect Concentration
EC-JRC: European Commission's Joint Research Centre
ESAC: EURL-ECVAM Scientific Advisory Committee
EU-NETVAL: European Union Network of Laboratories for the Validation of Alternative Methods
EURL-ECVAM: European Union Reference Laboratory on Alternatives to Animal Testing
GD: Guidance Document
GIVIMP: Guidance Document on Good In Vitro Method Practices
GL: Guideline
GLP: Good Laboratory Practice
GR: Glucocorticoid Receptor
h-CLAT: Human Cell Line Activation Test
HTS: High-Throughput Screening bioassays
IATA: Integrated Approach for Testing and Assessment
ISO: International Organization for Standardization
ITS: Integrated Testing Strategy
IVIVE: In Vitro To In Vivo Extrapolation
kDPRA: Kinetic Direct Peptide Reactivity Assay
KE: Key Event
MAD: Mutual Acceptance of Data
MIE: Molecular Initiating Event
MPS: Microphysiological Systems
NAM: New Approach Methodologies
OECD: Organisation for Economic Co-operation and Development
PARC: European Partnership for the Assessment of Risks from Chemicals
PC: Positive Control
PC10: The concentration inducing a 10% response in relation to the positive control
PEPPER: Public–private platform for the pre-validation of testing methods on endocrine disruptors
QSAR: Quantitative Structure–Activity Relationship
SOP: Standard Operation Procedure
TG: Test Guideline
TGP: Test Guideline Programme
US: United States (of America)
WNT: OECD Working Group of the National Coordinators to the Test Guideline Programme
