# Supplementary material for: Avoiding a reproducibility crisis in regulatory toxicology—on the fundamental role of ring trials
Source: Arch Toxicol. 2024 Apr 30;98(7):2047–63. doi: 10.1007/s00204-024-03736-z (PMC11169035; doi:10.1007/s00204-024-03736-z)
Supplement: Supplementary file 1 — Supplementary file1 (DOCX 20 KB) [file 204_2024_3736_MOESM1_ESM.docx]

# Supplementary information

# Case study on skin sensitization

The field of skin sensitization has maybe received most attention recently in terms of the number of new validated methods and OECD TGs. At the same time, it served as the poster child to set up the concept of adverse outcome pathways and it is the first endpoint, for which defined approaches (DA) were implemented in an OECD guideline subject to MAD—eventually putting NAM data on equal footing with the *in vivo* tests of a somewhat more complex endpoint (OECD 2023a, Guideline no. 497).

But the case of skin sensitization is also interesting for the discussion as to how test validation was conducted, and to illustrate the changing landscape.

The development of most NAMs for sensitization started by proposing the methods as stand-alone assays, with a prediction model to best discriminate sensitizers from non-sensitizers. Therefore, also the validation studies focused on reliability and predictive capacity of the yes/no answer. Later these methods were integrated as so-called information sources (alias the individual TGs addressing the skin sensitization key events) in the DA adopted as OECD Guideline no. 497 (OECD 2023a, Guideline no. 497). Nevertheless, the cell-based test methods, provided continuous data from the concentration-response curves. These data are available from the validation studies, but they were not part of the peer-review as conducted by ESAC- which evaluated the predictivity of the yes/no outcome according to the predefined prediction model only.

The 2o3 DA in GL 497 is a DA combining the outcomes of three test methods after the quantitative outcomes of each method has been dichotomised into a yes/no answer by thresholds which were established and validated for each method separately. The ITS DAs in GL 497 uses quantitative data and thresholds different from those originally defined for the individual methods adopted as TGs. Such situations, where only a final DA or ITS will define how the quantitative data from individual information sources is used, will become more frequent as it often represents the most effective approach for meaningful utilisation of NAMs. This trend is also seen with the newly emerging models to derive a Point of Departure for skin sensitization risk assessment such as different Bayesian models or quantitative regression models, which rely on the continuous quantitative data. To assess the experimental uncertainty of such models, the quantitative variability will be key, and it will require an assessment of the variability of quantitative data generated in ring trials. This underlines the importance of moving towards quantitative validation.
